# The society for immunotherapy of cancer consensus statement on immunotherapy for the treatment of advanced renal cell carcinoma (RCC)

**DOI:** 10.1186/s40425-019-0813-8

**Published:** 2019-12-20

**Authors:** Brian I. Rini, Dena Battle, Robert A. Figlin, Daniel J. George, Hans Hammers, Tom Hutson, Eric Jonasch, Richard W. Joseph, David F. McDermott, Robert J. Motzer, Sumanta K. Pal, Allan J. Pantuck, David I. Quinn, Virginia Seery, Martin H. Voss, Christopher G. Wood, Laura S. Wood, Michael B. Atkins

**Affiliations:** 1 Cleveland Clinic Taussig Cancer Center, Cleveland, OH USA; 2 KCCure, Leesburg, VA USA; 3 Cedars-Sinai Medical Center, Los Angeles, CA USA; 4 Duke University School of Medicine, Durham, NC USA; 5 UT Southwestern, Dallas, TX USA; 6 Charles A. Sammons Cancer Center, Baylor, Dallas, TX USA; 7 MD Anderson Cancer Center, Houston, TX USA; 8 Mayo Clinic, Jacksonville, FL USA; 9 Beth Israel Deaconess Medical Center, Boston, MA USA; 10 Memorial Sloan-Kettering Cancer Center, New York, NY USA; 11 City of Hope, Duarte, CA USA; 12 UCLA School of Medicine, Los Angeles, CA USA; 13 Norris Comprehensive Cancer Center, Los Angeles, CA USA; 14 Georgetown, Lombardi Comprehensive Cancer Center, Research Building, Room E501 3970 Reservoir Road NW, Washington, DC 20057 USA

**Keywords:** Guidelines, Immunotherapy, Renal cell carcinoma (RCC), Kidney cancer, Immune checkpoint inhibitor (ICI)

## Abstract

The approval of immunotherapeutic agents and immunotherapy-based combination strategies in recent years has revolutionized the treatment of patients with advanced renal cell carcinoma (aRCC). Nivolumab, a programmed death 1 (PD-1) immune checkpoint inhibitor monoclonal antibody, was approved as monotherapy in 2015 for aRCC after treatment with a VEGF-targeting agent. In April 2018, the combination of nivolumab and ipilimumab, a CTLA-4 inhibitor, was approved for intermediate- and poor-risk, previously untreated patients with aRCC. Then, in 2019, combinations therapies consisting of pembrolizumab (anti-PD-1) or avelumab (anti-PD-ligand (L) 1) with axitinib (a VEGF receptor tyrosine kinase inhibitor) were also approved to treat aRCC and are likely to produce dramatic shifts in the therapeutic landscape. To address the rapid advances in immunotherapy options for patients with aRCC, the Society for Immunotherapy of Cancer (SITC) reconvened its Cancer Immunotherapy Guidelines (CIG) Renal Cell Carcinoma Subcommittee and tasked it with generating updated consensus recommendations for the treatment of patients with this disease.

## Introduction

The development of novel immuno-oncology (IO) therapeutics has transformed the treatment paradigm for patients with advanced renal cell carcinoma (aRCC) and altered the role of previous approaches involving antiangiogenic agents targeting the vascular endothelial growth factor (VEGF) pathway. On November 23, 2015, the U.S. Food and Drug Administration (FDA) approved the anti-PD-1 monoclonal antibody nivolumab (Bristol Myers Squibb) for treatment of patients with aRCC after prior anti-angiogenic therapy [1]. On April 16, 2018 the FDA approved combination immunotherapy nivolumab (anti-PD-1) and ipilimumab (Bristol Myers Squibb; anti-cytotoxic T-lymphocyte-associated protein-4 [CTLA-4]) for the treatment of patients with intermediate or poor risk, previously untreated aRCC. Then, on April 19, 2019 and on May 14th, 2019, FDA approved pembrolizumab (Merck, Inc.; anti-PD-1) in combination with axitinib (Pfizer, Inc.; a VEGF receptor tyrosine kinase inhibitor; TKI) as well as avelumab (EMD Serono/Pfizer inc.; anti-PD-L1) in combination with axitinib, respectively, for the first-line treatment of patients with aRCC. Such approvals of first-line combination regimens will further expand and complicate RCC treatment options.

The advances in IO therapy over the past decade prompted the need to apply this knowledge to improve the management of patients with aRCC, including the emergence of IO in combination with TKIs, appropriate patient selection considerations, therapy sequencing, response monitoring, adverse event management, and biomarker application. In order to address these issues, the Society for Immunotherapy of Cancer (SITC) published the original *Renal Cell Carcinoma* clinical guidelines in November 2016 to provide evidence-based recommendations on how best to incorporate immunotherapies into practice for the treatment of patients with aRCC [2]. Recent advances in IO combinations have substantially added to the treatment approaches for patients with aRCC. To address these advances, the SITC *Cancer Immunotherapy Guidelines – Advanced Renal Cell Carcinoma* Subcommittee determined that the field would benefit from the production of an updated consensus recommendation*.* This panel - including expert physicians, nurses, scientists, and a patient advocate - regularly communicated via email, teleconference, and in-person between September 2018 and June 2019 to review existing new data and determine how to incorporate these results into an updated aRCC-specific consensus management guidelines. These resulting recommendations are meant to provide guidance to clinicians with the most up-to-date data and recommendations on how to best integrate immunotherapy into the treatment paradigm for patients with advanced RCC.

## Materials and methods

### Consensus statement policy

The National Academy of Medicine’s (NAM, formerly the Institute of Medicine) Standards for Developing Trustworthy Clinical Practice Guidelines reported in March 2011 were used as a model to generate this consensus statement [3]. In addition, methods applied previously to SITC consensus guidelines were used in order to develop and organize this manuscript [4]. As outlined by NAM, consensus guideline standards should include a transparent process for guideline development, funding sources, and the reporting and management of conflicts of interest accomplished by a multidisciplinary and balanced committee. The committee, nominated to establish an evidence-based foundation for recommendations and rating system to assess the strength of the evidence, reports the results through a peer-reviewed publication and publicly available website, and updates the statement as required by changes in the field. A draft of this consensus statement was made publicly available for comment between 8/12/2019 and 9/15/2019. The subcommittee should base its recommendations on evidence in the literature with a rating system to evaluate the strength of supporting peer-reviewed publications and results from reported clinical trials.

This consensus statement is intended to provide guidance and is not a substitute for the professional judgment of each individual treating physician and for each individual patient. Full consensus recommendations, for this disease as well as others, can be found on the SITC website [5]. Due to differences in drug approval, availability and regulations in some countries, this panel focused solely on United States FDA-approved drugs and regimens for the treatment of aRCC patients.

#### Cancer immunotherapy guideline – renal cell carcinoma subcommittee

The *Cancer Immunotherapy Guideline – Renal Cell Carcinoma* subcommittee consisted of nineteen participants, including thirteen medical oncologists, three urologists, one nurse, one nurse practitioner, and one patient advocate (Additional file 1). 100% of clinical subcommittee members reported previous experience/knowledge about the use IO therapy for the treatment of patients with aRCC. The subcommittee convened in February 2019 in accordance with the National Academy of Medicine and SITC processes to review guideline development progress as well as discuss the results from a previously distributed questionnaire collecting information on the participants’ role in the care of patients with aRCC and their current approach to various aspects of patient management. The clinical questionnaire addressed topics related to the role of the subcommittee members including primary clinical focus, experience with FDA-approved agents used for immunotherapy treatments, and current practices in the use or recommendation for use of such agents. The final consensus statement was made available to the entire SITC membership for open comment.

### Evidence and consensus ratings

Similar to the National Comprehensive Cancer Network (NCCN), SITC Cancer Immunotherapy Guidelines use categories of evidence. All recommendations are considered category 2A unless otherwise noted [6]. Consensus was defined as ≥75% agreement among SITC’s Cancer Immunotherapy Guidelines committee members.

### Consensus panel and conflicts of interest

In accordance with previous SITC practices used in development of consensus guidelines, nominated multidisciplinary subcommittee members were both SITC members and nonmembers who were expected to be affected by the development of clinical guideline recommendations including clinicians, patient representatives, nurses, and others. All subcommittee members were required to disclose any conflicts of interest using a SITC-specific disclosure form, mandating disclosure of full financial details and relationships with commercial entities that could be expected to have direct regulatory or commercial impact resulting from the publication of this statement. No commercial funding was provided to support the consensus subcommittee, literature review, or the preparation of this manuscript.

### Literature review process

The MEDLINE database was used to search the scientific literature for current therapies related to renal cell carcinoma and immunotherapy in humans and encompassed articles published from 2012 to 2019, including clinical trials, meta-analyses, practice guidelines, and research in humans. The search terms included “renal cell carcinoma OR RCC” and “ipilimumab”, “nivolumab”, “ipilimumab AND nivolumab”, “PD-1,” “PD-L1,” “CTLA-4”, “immunotherapy”, “immune checkpoint inhibitor”, “PD-1/PD-L1”, “combination therapy AND immunotherapy”, “immunotherapy AND biomarkers”, “adverse event”, “immunotherapy AND non-clear cell”, “pembrolizumab”, “ipilimumab”, and “toxicity”. Articles which were screened by subcommittee members to include only papers with clinically accurate and relevant information and to remove duplicates articles from independent searches, resulting in a final citation list catalogued using EndNote X7. The citation list was supplemented with additional articles identified by the panel, as appropriate and necessary for a comprehensive literature review.

### Consensus recommendations

Consistent with current FDA-approved immunotherapies, the *Cancer Immunotherapy Guideline – Renal Cell Carcinoma* subcommittee generated the following consensus recommendations for management of aRCC.

Traditional oncology clinical trials are generally designed to investigate one novel therapeutic agent or combination in comparison to a standard of care therapy. Cross-trial comparisons of two or more novel therapeutic strategies is hazardous, even with a common control arm, given the multiple potential variables in trial conduct including eligibility criteria, endpoints, patient management, timeframe, participating countries and institutions and availability of salvage therapies. This lack of statistical evidence comparing one specific approach to another in relation to their benefit beyond the standard of care poses an obvious limitation to the conclusions made by the subcommittee and to the development of the consensus recommendations provided.

## Key clinical questions

1. ***How should checkpoint inhibitors be integrated into the first-line treatment of advanced clear cell renal cell carcinoma (accRCC)?***

### General considerations

The integration of immunotherapeutic monoclonal antibodies directed against CTLA-4, PD-1, and PD-L1 - also known as IO agents - is now an essential part of the overall treatment strategy for patients with aRCC [7].

*The subcommittee would like to note, that even in the era of more active immune therapy, patients with aRCC and limited, indolent metastases can still be considered candidates for either initial observation, local approaches such as surgical resection of metastases and ablative techniques to metastases such as stereotactic body radiotherapy (SBRT) [8]. In general, suitable candidates for such an approach include those with a long time interval from primary tumor to development of metastases, slow growing disease and a limited number of metastatic sites.

### Literature review and first-line consensus recommendations

Table 1 describes major phase 3 trials investigating first-line IO-based therapies in patients with aRCC.

**Table 1:** Front-line, phase 3 immune checkpoint inhibitor-based trials in advanced RCC

Trial	Description	Results (combination vs. SOC), 95% CI		
		OS	PFS (months)	Objective Response
CheckMate 214 (NCT02231749) [9, 10]	Nivolumab + Ipilimumab vs. Sunitinib	ITT Population 12-mo: 83% vs. 77% 30-mo: 64% vs. 56% (HR 0.71; 0.59 to 0.86; P = 0.0003) I/P Risk Patients 12-mo: 80% vs. 72% 30-mo: 60% vs. 47% (HR 0.66; 0.54 to 0.80; P < 0.0001) Favorable Risk 12-mo: 94% vs. 96% 30-mo: NR vs. NR; (HR 1.22; 0.73 to 2.04)	ITT Population^a^ mPFS: 9.7 vs. 9.7 (HR 0.85; 0.73 to 0.98; P = 0.027, NS) I/P Risk Patients^a^ mPFS: 8.2 vs. 8.3 (HR 0.77; 0.65 to 0.90; P = 0.001, NS) Favorable Risk^a^ mPFS: 13.9 vs. 19.9 (HR 1.23; 0.90 to 1.69; P = 0.189, NS)	ITT Population^a^ 41% vs. 34%; (P = 0.0154, NS) CR: 11% vs. 2% I/P Risk Patients^a^ 42% vs. 29%; (P < 0.001) CR: 11% vs. 1% Favorable Risk^a^ ORR: 39% vs. 50%; (*P* = 0.1436) CR: 8% vs. 4%
Keynote-426 (NCT02853331) [11]	Pembrolizumab + Axitinib vs. Sunitinib	ITT Population 12-mo: 90% vs 78% (HR 0.53; 0.38–0.74; P < 0.0001)	ITT Population mPFS: 15.1 vs. 11.1 (HR 0.69; 0.57–0.84; P = 0.0001)	ITT Population ORR: 59% vs 36%; *P* < 0.0001) CR: 5.8% vs. 1.9%
Javelin RENAL 101 (NCT02684006) [12, 13]	Avelumab + Axitinib vs. Sunitinib	ITT Population 12-mo: 86% vs. 83% (HR 0.78; 0.55 to 1.08; p = 0.14)	ITT Population mPFS: 13.8 vs. 8.4 (HR 0.69; 0.56 to 0.84; P < 0.0001)	ITT Population ORR: 51% vs. 26% CR: 3.4% vs. 1.8%
IMmotion151 (NCT02420821) [14]	Atezolizumab + Bevacizumab vs. Sunitinib	ITT Population 24-mo: 63% vs. 60% (HR 0.93; 0.76 to 1.14; p = 0.4751)	ITT Population^a^ mPFS: 11.2 vs. 8.4 (HR 0.83; 0.70 to 0.97; p = 0.0219)	ITT Population ORR: 37% vs. 33%

^a^ Investigator-assessed, data not assessed by Independent Review Committee (IRC)

**Fig. 1:**
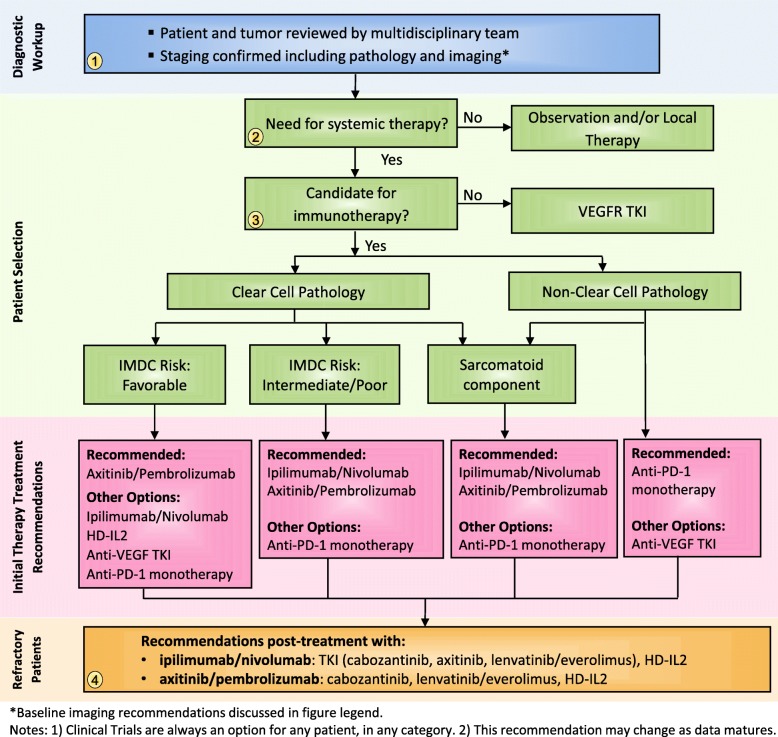
Immunotherapy treatment algorithm for patients with advanced renal cell carcinoma

Formerly, patients with aRCC primarily received sequential monotherapy with TKIs and possibly mTOR-targeted therapy [1]. A subset of patients with mRCC were also able to receive treatment with high dose IL-2 which produced durable responses (CRs and some PRs) in a small subset of patients; however the majority of patients were not able to receive this treatment due to its potential toxicity, complexity and thus limited availability [15]. In 2015, single-agent nivolumab became available in the second-line treatment of aRCC and paved the way for future immunotherapy regimens. Based on the results of CheckMate 214, Keynote-426 and other phase III combination therapy trials, IO and IO/TKI strategies have changed the treatment paradigm for patients with aRCC [Fig. 1. Treatment Algorithm] [9, 16].

Regarding whether IMDC categories are still relevant for treatment decision making in light of the development of the IO-based combination therapy regimens in determining whether or not to recommend anti-PD-1 combination therapy, 59% of subcommittee members felt they were not relevant, while 41% felt that they still provided information that might influence treatment choice. Distinctly, in determining whether or not to recommend anti-PD-1/TKI combination therapy, 76% of subcommittee members felt they were not relevant, while 24% felt that they still provided information that might influence treatment choice. Regardless of their clinical decision making utility, most subcommittee members thought the categories were still useful in assessing prognosis and for stratifying patients in clinical trials. Feedback from the patient advocacy community suggested that going forward, when using IMDC risk criteria in decision making with patients, the words “poor” risk and “favorable” risk should either be replaced with high risk and low risk or the community should move toward IMDC groups 1, 2, and 3., The majority of the subcommittee (74%) routinely order laboratory and other tests to determine IMDC risk group stratification prior to treatment of patients with newly diagnosed accRCC.

In addressing preliminary issues surrounding frontline management of RCC, the subcommittee recommends initiating systemic therapy first rather than cytoreductive nephrectomy in patients presenting with metastatic RCC with: IMDC poor risk categorization (80% of committee members), brain metastases (67%) or a large tumor burden outside primary kidney lesion (60%) [17]. Cytoreductive nephrectomy is still considered a preferable option for patients with the majority of of their tumor burden confined to their primary and no other IMDC risk factors besides presenting with stage IV disease.

### Specific agents and combinations

Very little data exists regarding nivolumab monotherapy for first-line treatment of patients with aRCC [18]. However, the randomized phase 3 CheckMate 214 trial examined nivolumab plus ipilimumab combination therapy followed by nivolumab monotherapy, compared to sunitinib monotherapy in previously untreated patients with accRCC [9, 10]. Patients received either nivolumab (3 mg/kg) plus ipilimumab (1 mg/kg) intravenously every three weeks for up to four doses, followed by nivolumab (3 mg/kg) every two weeks, or sunitinib (50 mg) orally once per day for four weeks, during a 6-week cycle (Sunitinib 50 mg 4/2). This dosing schedule was derived from the previous CheckMate016 study finding that this dosing was better tolerated than ipilimumab 3 mg/kg plus nivolumab 1 mg/kg with equal efficacy in patients with previous VEGF pathway therapy [5]. Outcomes were stratified according to the International Metastatic Renal Cell Carcinoma Database Consortium (IMDC), a validated model which categorizes the prognosis of patients with aRCC according to favorable-, intermediate-, or poor-risk disease depending on the presence of well-characterized clinical and laboratory risk factors [19, 20]. Primary endpoints included OS, ORR and PFS [9] in IMDC intermediate or poor-risk patients (I/P; *n* = 847) although the trial included subjects in all risk categories [20]. The trial demonstrated statistically significant improvements in OS and ORR for patients receiving the combination compared with those receiving sunitinib that persisted at the 30-month follow-up (Table 2). Among the responders, 52% of patients receiving the immunotherapy combination experienced a response duration ≥18 months compared with 28% of patients treated with sunitinib. Of note, reported ORR, CR rate and response durability data from the 30-month follow-up in favorable risk patients treated with nivolumab/ipilimumab, detailed in Table 3, suggest that exclusion of favorable risk patients from the potential long term benefits of IO therapy may not be justified. That is, the subset of patients with durable CR or significant PR may justify consideration of ipilimumab plus nivolumab although the hazard ratios for OS, PFS and the ORR numerically, but no longer significantly, favor sunitinib for the entire favorable risk cohort. Importantly, patients reported better health-related quality of life (as measured by the FKSI-19) with nivolumab plus ipilimumab compared to those treated with sunitinib [9].

More recently, combinations of antiangiogenic agents with immunotherapeutic strategies have been evaluated. The biologic rationale for these combinations stems from preclinical studies in models which involved either non-clear cell tumors (e.g. RENCA) or other types of cancer altogether but which suggested that anti-VEGF agents could enhance antitumor immunity by increasing antigen presenting cell function, enhancing immune cell tumor infiltration, and decreasing effect of myeloid derived suppressor cells and macrophages in the tumor microenvironment [21].

IMmotion150 (NCT01984242), a randomized phase 2 study of atezolizumab (anti-PD-L1) monotherapy or in combination with bevacizumab (anti-VEGF antibody) versus sunitinib was investigated in 305 patients with treatment-naive cRCC. After progression on atezolizumab or sunitinib, crossover to atezolizumab/bevacizumab was allowed. Reported PFS hazard ratios for ITT patient population treated with atezolizumab/bevacizumab or atezolizumab monotherapy versus sunitinib were 1.0 (95% confidence interval (CI), 0.69–1.45) and 1.19 (95% CI, 0.82–1.71), respectively. After first-line treatment, 78% of patients treated with sunitinib and 60% of patients treated with atezolizumab who progressed later received atezolizumab/bevacizumab and achieved ORRs of 28 and 24%, respectively. Subsequently, the IMmotion151 (NCT02420821) phase 3 trial investigated the combination of atezolizumab with bevacizumab, compared to sunitinib [14]. Atezolizumab was administered at 1200 mg + bevacizumab at 15 mg/kg IV every 3 weeks or sunitinib at 50 mg 4/2. Primary endpoints included PFS in PD-L1+ patients (Table 3; ≥1% tumor-infiltrating immune cells [IC]) and OS in intent-to-treat (ITT) patients. Median survival follow-up was 15 months. PFS benefit was improved in the combination arm compared to sunitinib; however, no OS benefit was observed (Tables 1 and 3) [14].

An open-label, phase Ib, single-arm clinical trial investigated the combination of axitinib, a small molecule tyrosine kinase inhibitor TKI, and pembrolizumab in 52 treatment-naïve patients with aRCC. Median PFS was 20.9 months (95% CI, 15.4 to not evaluable) and an ORR of 73.1% was reported, including CRs in 7.7%, suggestive of substantial antitumor activity [22].

The phase 3 KEYNOTE-426 (NCT02853331) clinical trial further examined the combination of pembrolizumab with axitinib compared to sunitinib in patients with previously untreated accRCC. 861 patients were randomly assigned to receive pembrolizumab at a dose of 200 mg intravenously every three weeks for up to 35 doses plus axitinib 5 mg orally twice daily, or sunitinib (50 mg 4/2). At 12.8-month median follow-up, 59.0% of patients in the pembrolizumab/axitinib arm and 43.1% in the sunitinib arm remained on treatment. OS, PFS, and ORR benefits were observed with the combination across all risk groups and PD-L1 expression levels (Table 4) [11]. Results of this study mark the first time that a treatment improved endpoints of OS, PFS and ORR as frontline therapy in aRCC across all risk groups.

Parallel with previously reported phase 1 pembrolizumab/axitinib data, phase 1 data for the Javelin Renal 100 (NCT02493751) study demonstrated a manageable safety profile with encouraging antitumor activity [23]. Successively, the JAVELIN Renal 101 (NCT02684006) phase 3 study investigated the combination of avelumab (anti-PD-L1) with axitinib in 886 previously untreated patients with aRCC. Avelumab was administered at 10 mg/kg IV every two weeks in combination with axitinib, 5 mg orally twice daily. Sunitinib was given at 50 mg 4/2. Median PFS was improved in the combination arm compared to the sunitinib arm in the overall population, irrespective of risk factor and PD-L1 status (Tables 1, and 2) [12]. However, no overall survival benefit has been demonstrated for this combination. Specifically, at a median follow-up for overall survival of 11.6 months and 10.7 months, among the patients with PD-L1–positive tumors, deaths from any cause were observed in 37 patients (13.7%) who received avelumab plus axitinib and in 44 patients (15.2%) who received sunitinib (HR, 0.82; 95% CI, 0.53 to 1.28; *p* = 0.38). The median follow-up was 11.6 months and 10.7 months, respectively. In the overall population, HR for death for the two groups was 0.78 (95% CI, 0.554, 1.084; *p* = 0.14).

**Table 2:** Phase 3 immune checkpoint inhibitor-based adjuvant therapy trials in advanced RCC

Trial	Description	Primary Outcome to be Assessed
CheckMate 914 (NCT03138512)	Nivolumab + ipilimumab vs. placebo as adjuvant therapy in patients with localized RCC who underwent radical or partial nephrectomy and who are at high risk of relapse	Blinded Independent Central Review (BICR)-assessed disease-free survival (DFS)
IMmotion010 (NCT03024996)	Atezolizumab vs. placebo as adjuvant therapy for 1 year in patients with RCC at high risk of disease recurrence following nephrectomy	Independent review facility (IRF)-assessed DFS.
KEYNOTE 564 (NCT03142334)	Pembrolizumab vs. placebo (saline solution) as adjuvant therapy given after nephrectomy on 3-week cycles for up to 17 cycles in patients with resected intermediate or high risk ccRCC	Safety and efficacy and investigator-assessed DFS.
PROSPER RCC (NCT03055013)	Perioperative nivolumab vs. nephrectomy alone in treating patients with high-risk RCC	Recurrence-free survival (RFS).
RAMPART (NCT03288532)	Durvalumab monotherapy vs. durvalumab + tremelimumab vs. no intervention (active monitoring) as adjuvant therapy for 1 year in patients with resected primary RCC at high or intermediate risk of relapse	DFS and OS.

**Table 3:** Immune-related toxicity data reported in front-line combinations with ICIs in advanced RCC Clinical Trials

		CheckMate 214 Nivolumab + Ipilimumab (NCT02231749) [9] (*n* = 547)	Keynote-426 Pembrolizumab + Axitinib (NCT02853331) [11] (*n* = 429)	Javelin RENAL 101 Avelumab + Axitinib (NCT02684006) [12, 13] (*n* = 434)	IMmotion151 Atezolizumab + Bevacizumab (NCT02420821) [14] (*n* = 451)	CheckMate 025 Nivolumab (NCT01668784) [16] (*n* = 406)
Number of patients (%)						
Total Events	Any TRAE	509 (93)	422 (98.4)	432 (95.4)	411 (91)	319 (79)
	Grade 3–4 TRAEs	250 (46)	270 (63)	309 (56.7)	182 (40)	76 (19)
	TRAEs leading to discontinuation of either drug	–	131 (30.5)	–	Atezolizumab: 9 (2); Bevacizumab: 23 (5)	31 (8)
	TRAEs leading to discontinuation of both drugs	118 (22)	45 (10.7)	33 (7.6)	24 (5)	–
	Treatment related deaths	8 (1.5)	4 (0.9)	3 (0.7)	5 (1.1)	0
Most Common AEs (Any Grade)	Fatigue	202 (37)	165 (38.5)	180 (41.5)		134 (33)
	Pruritus	154 (28)		61 (14.1)		57 (14)
	Diarrhea	145 (27)	233 (54.3)	270 (62.2)		50 (12)
	Hypertension	12 (2)	191 (44.5)	215 (49.5)		–
	Rash	118 (22)	61 (14.2)	62 (14.3)		41 (10)
	Nausea	109 (20)	119 (27.7)	148 (34.1)		57 (14)
	Increased lipase	90 (16)	–	–		–
	Hypothyroidism	85 (16)	152 (35.4)	108 (24.9)		–
	Palmar-plantar erythrodysesthesia	5 (< 1)	120 (28.0)	145 (33.4)		–
Most Common TRAEs (Grade 3–4)	Fatigue	23 (4)	12 (2.8)	15 (3.5)		10 (2)
	Diarrhea	21 (4)	39 (9.1)	29 (6.7)		5 (1)
	Hypertension	4 (< 1)	95 (22.1)	111 (25.6)	63 (14)	–
	Increased lipase	56 (10)	–	–		–
	Palmar-plantar erythrodysesthesia	0	22 (5.1)	25 (5.8)		–
	Alanine aminotransferase increased	–	57 (13.3)	26 (6.0)		–
	Aspartate aminotransferase increased	–	30 (7.0)	17 (3.9)		–

**Table 4:** Biomarker data reported with ICIs in advanced RCC

Trial	Description	Results (combination vs. SOC), 95% CI		
		OS	PFS (months)	Objective Response
CheckMate 025 (NCT01668784) [16]	Nivolumab vs. Everolimus	PD-L1 ^τ^ (< 1%) ^a^ mOS: 27.4 vs. 21.2 mo (HR 0.77; 0.60 to 0.97) PD-L1 ^τ^ (≥1%) ^a^ mOS: 21.8 vs. 18.8 mo (HR 0.79; 0.53 to 1.17		
CheckMate 214 (NCT02231749) [9]	Nivolumab + Ipilimumab vs. Sunitinib	PD-L1 ^τ^ (< 1%) ^a^ HR 0.73; 95% CI, 0.56 to 0.96 12-mo rate: 80% vs. 75% 18-mo rate; 74% vs. 64% PD-L1 ^τ^ (≥1%) ^a^ HR 0.45; 0.29 to 0.71 12-mo rate: 86% vs. 66% 18-mo rate: 81% vs. 53% Sarcomatoid Histology HR 0.56; 0.38–0.83 PD-L1 (≥1%) prevalence: 50% (SH) vs. 27.5% (non-SH)	PD-L1 ^τ^ (< 1%) ^a^ mPFS: 11.0 vs. 10.4 (HR 1.00; 0.8 to 1.26) PD-L1 ^τ^ (≥1%) ^a^ mPFS: 22.8 vs. 5.9 (HR 0.46; 0.31 to 0.67)	PD-L1 ^τ^ (< 1%) ^a^ ORR: 37% vs. 28%, p = 0.03 PD-L1 ^τ^ (≥1%) ^a^ ORR: 58% vs. 22%, p < 0.001 Sarcomatoid Histology ORR: 56.7% vs. 19.2%, P < .0001 CR: 18.3% vs. 0%
Keynote-426 (NCT02853331) [11] [insert ASCO abstract 4500]	Pembrolizumab + Axitinib vs. Sunitinib	PD-L1 ^β^ (< 1%) ^b^ HR 0.59; 0.34 to 1.03 PD-L1 ^β^ (≥1%) ^b^ HR 0.54; 0.35 to 0.84 Sarcomatoid Histology 12-mo OS: 83.4% vs 79.5% (HR 0.58; 0.21 to 1.59)	PD-L1 ^β^ (< 1%) ^b^ HR 0.87; 0.62 to 1.23 PD-L1 ^β^ (≥1%) ^b^ HR 0.62; 0.47 to 0.80 Sarcomatoid Histology mPFS: NR vs. 8.4 (HR 0.54; 0.29 to 1.00)	Sarcomatoid Histology ORR: 58.8% vs 31.5% CR: 11.8% vs. 0%
Javelin RENAL 101 (NCT02684006) [12, 13]	Avelumab + Axitinib vs. Sunitinib	Not available.	PD-L1^¥^ + (≥1%) ^c^ mPFS: 13.8 vs 7.2 (HR 0.61; 0.47 to 0.79; P < 0.001) PD-L1^¥^- (< 1%) ^c^ mPFS: 16.1 vs. 11.1	PD-L1^¥^ + (≥1%) ^c^ ORR: 55.2% vs 25.5% CR: 4.4% vs. 2.1% PD-L1^¥^- (< 1%) ^c^ ORR: 47% vs. 28%
IMmotion150 [46]	Atezolizumab + Bevacizumab or Atezolizumab monotherapy vs. Sunitinib	Not available.	ITT Population Combination HR 1.0; 0.69 to 1.45 Monotherapy HR 1.19; 0.82 to 1.71 PD-L1^¥^ + (≥1%) ^d^ Combination HR 0.64; 0.38 to 1.08 Monotherapy HR 1.03; 0.63 to 1.67	PD-L1^¥^ + (≥1%) ^d^ 48% (combination) and 28% (monotherapy) vs. 27%
IMmotion151 (NCT02420821) [14] [insert ASCO abstract citation]	Atezolizumab + Bevacizumab vs. Sunitinib	PD-L1^¥^ + (≥1%) ^d^ OS: 75% vs. 65% (HR 0.68; 0.46–1.0; p = 0.0470) Sarcomatoid Histology All Sarc mOS: NR vs. 15.0 (HR: 0.56; 0.32 to 0.96) 12-mo OS: 69% vs. 60% PD-L1+ Sarc mOS: NR vs. 15.0 (HR: 0.53; 0.27 to 1.06) 12-mo OS: 71% vs. 61%	PD-L1^¥^ + (≥1%) ^d^ mPFS: 11.2 vs. 7.7 (HR 0.74; 0.57 to 0.96; p = 0.0217) Sarcomatoid Histology All Sarc mPFS: 8.3 vs. 5.3 (HR: 0.52; 0.34 to 0.79) PD-L1+ Sarc mOS: 8.6 vs. 5.6 (HR: 0.45; 0.26 to 0.77) Gene Expression Signatures High T_eff_ mPFS: 12.45 vs. 8.34 (HR 0.76; 0.59–0.99) Low T_eff_ mPFS: 9.72 vs. 8.41 m High angiogenesis mPFS: 12.45 vs. 10.2 (HR 0.95; 0.75–1.19) Low angiogenesis mPFS: 8.94 vs. 5.95 (HR 0.68; 0.52–0.89)	PD-L1^¥^ + (≥1%) ^d^ ORR: 43% vs 35% Sarcomatoid Histology All Sarc ORR: 49% vs. 14% CR: 10 vs. 3% PD-L1+ Sarc ORR: 56% vs. 12% CR: 14% vs. 4%

*SH* Sarcomatoid Histology

Cell population used: ^τ^ = tumor cells, ^¥^ = immune cells, ^β^ = both tumor and immune cells

Antibody used: ^a^ = Rabbit 28–8 (Dako), ^b^ = Mouse 22C3 (pharmDx), ^c^ = Rabbit SP263 (Ventana), ^d^ = Rabbit SP142 (Ventana)

Of the possible combination therapies including a VEGF inhibitor combined with an immune checkpoint inhibitor, 94% of the subcommittee recommended pembrolizumab plus axitinib as the preferred combination for patients with aRCC.

For a treatment naïve, ECOG 0 ccRCC patient with “favorable” risk per IMDC, who is determined to need systemic therapy and has no contraindication to receiving either an IO or an anti-VEGF therapy, 50% of the subcommittee recommend treatment with axitinib/pembrolizumab, 28% recommend treatment with nivolumab/ipilimumab, 11% recommend TKI monotherapy, and 6% recommend treatment with either axitinib/avelumab or HDIL-2.

For a treatment naïve, ECOG 0 ccRCC patient with “intermediate/poor” risk per IMDC, who is determined to need systemic therapy and has no contraindication to receiving either an IO or an anti-VEGF therapy, 78% recommend treatment with nivolumab/ipilimumab, 17% of the subcommittee recommend treatment with axitinib/pembrolizumab, and 6% recommend ICI monotherapy.

Anti-PD-1 monotherapy has also been tested as first-line therapy in patients with accRCC. Results from cohort A of the phase 2 KEYNOTE-427 trial (NCT02853344) investigating pembrolizumab (anti-PD-1) monotherapy as first-line therapy for the treatment of patients with accRCC were presented at the 2018 American Society of Clinical Oncology (ASCO) Congress [24]. Pembrolizumab was administered at a dose of 200 mg intravenously every three weeks for two years or until confirmed progressive disease (PD), unacceptable toxicity, or patient withdrawal. Median follow-up was 7.2 (0.9–11.7) months at the data cutoff (October 6, 2017). Of 107 patients, 37.3, 47.3, and 15.5% had IMDC risk categories of favorable, intermediate, and poor, respectively. Confirmed ORR was 38.2% (*n* = 42; 95% CI, 29.1–47.9) with 3 CR (2.7%) and 39 (35.5%) PRs in the overall patient population. ORR for patients with favorable, intermediate/poor risk IMDC was 31.7 and 42%, respectively. Median duration of response (DOR) was not reached (range, 1.4+ to 8.2+) [24]. Checkpoint inhibitor monotherapy, however, has not yet been approved by regulatory authorities or tested in a randomized study, and thus the precise role requires further investigation.

In determining when to give a treatment-naïve patient IO monotherapy over an IO-based doublet therapy, the subcommittee recommend IO monotherapy for patients with a history of autoimmune disease that is not potentially life threatening and is not currently on immunosuppressive agents (56%), elderly patients over 80 years of age (50%), patients with a history of vascular disease such as stroke, recent ischemic cardiac disease without CABG (39%), patients with poor performance status (28%), patients with IMDC favorable risk (6%), and patients with liver metastases with mildly increased LFTs (6%). 17% of subcommittee members would never recommend IO monotherapy over an IO-based doublet therapy.

Given the current data, the subcommittee felt that all patients without a contraindication to immunotherapy should receive an IO-based regimen in the first line. Contraindications to anti-PD1 therapy include active or a history of life threatening autoimmune conditions and the requirement for corticosteroids (> 10 mg prednisone equivalent) for treatment of cancer-related conditions. Additionally, disease progression within 6 months of an adjuvant immunotherapy regimen was felt to be a potential contraindication, although the activity of IO-based doublets in this setting are unknown.

2. ***How should checkpoint inhibitors be integrated into treatment of refractory accRCC?***

In 2015, backed by the results of the CheckMate 025 trial (NCT01668784), nivolumab gained FDA approval for the treatment of patients with aRCC who have received prior antiangiogenic therapy [1]. While this second-line therapy approval changed the treatment landscape for patients with aRCC previously treated with VEGFR TKIs, there exists considerable uncertainty and limited data as to how to treat patients with aRCC who have progressed on more recently approved first-line IO-based combination therapies.

### Literature review and second-line consensus recommendations

Category 1 evidence is provided in data from CheckMate 025 for use of single agent anti-PD-1 immunotherapy for patients with accRCC who were previously treated with a VEGFR TKI.

The randomized phase 3 CheckMate 025 study compared nivolumab to everolimus as therapy in previously treated patients with RCC. In this study, patients received either 3 mg/kg of nivolumab intravenously every two weeks or 10 mg everolimus orally once per day. Median overall survival (OS) for nivolumab compared to everolimus was 25.0 months (95% confidence interval [CI], 21.8 to not estimable [NE]) and 19.6 months (95% CI, 17.6 to 23.1), respectively. The hazard ratio (HR) for death was 0.73 (98.5% CI, 0.57 to 0.93; *P* = 0.002). The objective response rate (ORR) was greater in patients treated with nivolumab compared to everolimus (25% vs. 5%; odds ratio, 5.98 [95% CI, 3.68 to 9.72]; *P* < 0.001). Median progression-free survival (PFS) for nivolumab versus everolimus was 4.6 months (95% CI, 3.7 to 5.4) and 4.4 months (95% CI, 3.7 to 5.5; HR = 0.88, 95% CI, 0.75 to 1.03; *P* = 0.11), respectively [16]. The role of nivolumab monotherapy is evolving given that nivolumab plus ipilimumab is now a front-line standard, the approval of other PD-1 pathway based combinations (see above), and thus fewer patients will be receiving nivolumab monotherapy.

Nivolumab was initially investigated in combination with CTLA-4 antibodies in patients with mRCC, approximately half of whom had received prior therapy, as part of the CheckMate 016 study. Confirmed ORR was seen in 36.2 and 40.4% of patients, respectively with either nivolumab 3 mg/kg + ipilimumab 1 mg/kg (N3I1 arm) or nivolumab 1 mg/kg + ipilimumab 3 mg/kg (N1I3 arm) regimen [5, 10]. Median PFS was 7.0 and 9.4 months for each regimen, respectively. Follow-up data suggested that over 50% of patients were alive and free from subsequent therapy at 3 years [25]. This data support the combination of nivolumab/ipilimumab as salvage therapy after prior VEGFR therapy (see below for data related to second-line therapy).

A small-scale retrospective analysis of patients treated with HD IL-2 following disease progression after PD-1 or PD-L1 inhibitor treatment showed that prior checkpoint inhibitor therapy may not be detrimental to subsequent treatment with HD IL-2 in patients with RCC. Of 17 patients with mRCC who previously received PD-1 or PD-L1 inhibitors, there were 4 responses (2 complete, 2 partial) to HD-IL-2 therapy and the toxicity profile was similar to that seen in patients receiving front-line HD IL-2 [26].

For a previously treated, ECOG 0, clear cell mRCC patient with “favorable” risk whose tumors progressed on front-line therapy with sunitinib, 100% of the subcommittee recommend treating with a checkpoint immunotherapy but were split in 37/63% by nivolumab monotherapy versus ipilimumab plus nivolumab combination immunotherapy if the patient can tolerate. Of note, as standard of care shifts to immunotherapy regimens in the first line setting, this situation will be unlikely to occur in the future and the use of VEGFR TKI monotherapy as first-line therapy will be limited to those patients who are perceived to be unable to be receive a checkpoint inhibitor based treatment regimen.

In treating patients with disease progression after nivolumab/ipilimumab combination therapy, 72% of the subcommittee recommend treatment with cabozantinib, 22% recommend axitinib and 6% recommend HD IL-2.

In treating patients with disease progression after IO/VEGFR TKI combination therapy (either axitinib/pembrolizumab or axitinib/avelumab), the subcommittee consensus was to recommend treatment with cabozantinib (83%), while 11% recommended nivolumab/ipilimumab and 6% recommended lenvantinib/everolimus.

Specifically, the subcommittee also acknowledged that no data existed for the use of nivolumab/ipilimumab in patients with disease progression on an IO/TKI combination or for the use of a IO/TKI combination in patients with disease progression on front-line nivolumab/ipilimumab, and suggested that clinical trials to obtain such data would be useful.

3. ***How should adjuvant therapy and related failures be managed within an IO-related treatment paradigm for patients with accRCC?***

With sunitinib approved in the adjuvant setting based on data from the S-TRAC trial and the widespread use of IO therapy in ongoing adjuvant and neoadjuvant trials, questions regarding management strategies arise [27]. Issues include the risk of potentially permanent side effects (diabetes, immune related arthritis, etc.) associated with IO, especially important after potentially curative surgery, the duration of treatment, and the choice of therapy in patients who have received various prior adjuvant treatments.

### Literature review and consensus recommendations

Several phase III adjuvant therapy trials are ongoing in the treatment of RCC (Table 2).

#### SOC in the adjuvant setting (ongoing trials)

In determining which factors would influence their recommendation against treating patients with advanced RCC with combination IO, 67% of the subcommittee would recommend nivolumab/ipilimumab to a patient with aRCC who received prior adjuvant IO therapy within the last 6 months (33% of the subcommittee would choose not to recommend nivolumab/ipilimumab in this setting). Similarly 67% of the subcommittee would recommend IO/TKI therapy to a patient with advanced RCC who had previously received either adjuvant IO or adjuvant sunitinib therapy within the last 6 months (33% of the subcommittee would choose not to recommend IO/TKI therapy in this setting).

In patients whose disease has progressed at or beyond 6 months following adjuvant anti-PD-1/PD-L1 monotherapy, the subcommittee was split (47%/47%) as to their recommendation of an IO/IO or IO/TKI regimen following adjuvant immunotherapy, specifically nivolumab/ipilimumab vs. axitinib/pembrolizumab.

In patients whose disease has progressed > 6 months following completion of adjuvant sunitinib, the majority of the subcommittee (93%) recommends treatment with nivolumab/ipilimumab combination therapy.

4. ***How should immune-related adverse events be recognized and managed in patients with accRCC?***

Patients treated with immunotherapy have demonstrated specific side effects known as immune-related adverse events (irAEs). Overall, monoclonal antibodies targeting checkpoint proteins have a different and less predictable toxicity profile than VEGFR TKIs [28–34]. Although 30–40% of patients can have severe toxicities from nivolumab/ipilimumab requiring a course of corticosteroids and/or other immunosuppressive agents, many patients have minimal side effects from IO therapy. However, irAEs are consistently reported and can affect any organ system, including but not limited to manifestations such as colitis, pneumonitis, endocrinopathies, or hepatitis [28, 29, 31, 35, 36]. Additional management considerations in patients with aRCC may include the occurrence of nephritis in patients with a single kidney. While complete management recommendations are outside the context of this manuscript, the subcommittee discussed general irAE management strategies in patients with aRCC.

### Literature review and consensus recommendations

All studies discussed below (Table 2.) were graded according to the National Cancer Institute Common Terminology Criteria for Adverse Events (CTCAE), version 4.0.

#### Toxicity management consensus recommendations

The subcommittee discussed when to change clinical management of patients treated with IO therapies based on irAEs. The subcommittee felt that kidney cancer management of irAEs is aligned with the management of these toxicities in other solid tumor and provided recommendations concerning when, at which grade of toxicity, and for which adverse events to hold therapy. For further detail into toxicity management strategies please refer to ASCO’s Management of Immune-Related Adverse Events in Patients Treated with Immune Checkpoint Inhibitor Therapy: American Society of Clinical Oncology Clinical Practice Guideline.

The subcommittee was split in deciding when to hold PD-1 based monotherapy (including during the maintenance component of the nivo/ipi regimen) due to irAEs. Fifty percent recommended not holding treatment unless it is a grade 3 toxicity, while 50% supported holding of therapy for patients with some worrisome grade 2 toxicities (diarrhea, arthritis, dyspnea, hepatitis, etc). Another reason to hold PD-1 monotherapy included occurrence of multiple grade 2 toxicities.

Regarding how to best manage clinically-significant grade 3 irAEs in patients with accRCC receiving PD-1 based monotherapy (excluding endocrinopathies stable on replacement), the majority of the subcommittee (72%) advised holding therapy and starting oral high dose (HD) steroids and tapering over 4–6 weeks once symptoms resolve.

For a patient with stable disease or better on scans who has stopped induction therapy with nivolumab/ipilimumab due to a grade 3 or higher irAE, the subcommittee is split 50/50% in their recommendation to either wait until toxicity is ≤ grade 1 and the patient is taking prednisone at a dose of 10 mg/d or less and then begin anti-PD-1 monotherapy maintenance versus observing the patient while off all therapy until progression. No member supported the concept of resuming therapy while the patient was still on steroid therapy > 10 mg of prednisone equivalent per day.

Regarding when to hold nivolumab/ipilimumab combination therapy due to any grade irAEs, the majority of the subcommittee (67%) recommends to hold nivolumab/ipilimumab for grade 2 toxicities, treat with immunosuppressive drugs if they do not resolve, and resume with nivolumab monotherapy when/if the toxicities resolve, while a substantial minority of the subcommittee (27%) recommends to hold treatment for grade 1 or 2 toxicities (diarrhea, arthritis, LFT abnormalities) to see if they worsen before resuming.

Regarding when to hold IO/TKI combination therapy due to grade 3 toxicity (e.g. diarrhea, LFT abnormalities) that could be from either drug, the subcommittee recommends to hold axitinib for 2–3 days to see if toxicity improves (56%), hold both drugs and give steroids (22%), hold both drugs to see if toxicity improves (17%) or give steroids and hold the IO component, but continue axitinib (6%).

Regarding when to hold IO/TKI combination therapy due to any grade irAEs, the subcommittee was split in their recommendation of either holding axitinib treatment for grade 1 or 2 toxicities (diarrhea, arthritis, LFT abnormalities) to see if they worsen before resuming (60%) or to recommend not to hold treatment unless the patient is experiencing a Grade 3 toxicity (33%).

The majority of the subcommittee agreed the best way to educate patients on potential risks and side effects of immunotherapy was by meeting with the patient plus the patient’s family in office visits and giving the patient literature/guidelines to read. The subcommittee recommends that patients should be provided with literature in the doctor’s office (or online resources) to learn more fully about how immunotherapy works, what kinds of treatments and trials are available, and what their experience of treatment might be like, including toxicities. Given the less predictable toxicity profile of IO therapy, patients should have clear guidance and instructions on when to contact their provider to report symptoms to help protect against development of grade 3 AEs.

5. ***How should treatment response to immunotherapy be evaluated, monitored and managed in patients with accRCC?***

With the many new IO treatment regimens available comes the need to better understand patient monitoring and management strategies, including testing prior to immunotherapy administration, when to hold or delay treatment in the event of an irAE, for how long to continue treatment, and when to treat beyond progression.

Response kinetics following treatment with IO differs from those with molecularly targeted or cytotoxic agents. Treating physicians should be aware that non-linear response patterns may occur during and post-treatment with immunotherapy. For instance, pseudo-progression, defined as an initial flare of tumor size (suggestive of tumor progression) followed by a reduction in tumor mass is considered an uncommon, but possible, event in solid tumors [37, 38]. However, it should be noted that most progression is real and requires a change in therapy regimen.

As such, new methods of disease evaluation and surveillance have been developed, including IO-based response metrics, such as the immune-related response criteria (irRC) and immune-related Response Evaluation Criteria in Solid Tumors (iRECIST) [38, 39]. Based on these considerations, subcommittee members discussed optimal metrics with which to evaluate the clinical benefit of immunotherapy, how best to use radiographic response criteria such as RECIST, and time intervals for imaging evaluation of IO efficacy in order to prevent premature withdrawal of a potentially effective therapy for patients with aRCC.

### Literature review and consensus recommendations

All studies demonstrating efficacy of anti-PD-1 and anti-CTLA-4 have used RECIST v1.1 and this version continues to be used in most current immunotherapy clinical trials [40].

Traditional response evaluation by RECIST considers a significant (≥20%) increase in the size of tumor lesions and/or the development of new lesions to be explicit evidence of disease progression. However, tumors treated with immunotherapy do not follow the same response patterns as those treated with chemotherapy and targeted treatments and immunotherapy-based response patterns such as tumor flare would be viewed as disease progression and may lead to premature discontinuation of treatment. Therefore, as some patients may benefit from continued immunotherapy beyond RECIST-defined first progression.

One study analyzed immune-modified response evaluation criteria in solid tumors (imRECIST) to assess its added value in capturing cancer immunotherapy responses. The study examined atezolizumab data from clinical trials and analyzed modifications made in developing imRECIST from RECIST v1.1. Such modifications included allowance for best overall response after PD as well as changes in PD definitions as new lesions and non-target lesions arise. RECIST v1.1 was modified so that PFS by imRECIST did not count initial PD as an event if subsequent scans showed disease control. OS was evaluated using conditional landmarks in patients whose PFS differed by imRECIST versus RECIST v1.1 Overall, immune-based response criteria appear more suitable for evaluation of immunotherapy [39, 41–43].

Although evaluation of patient response to immunotherapy still relies on RECIST criteria for reporting endpoints, immune-related response criteria (irRC) are being recognized as better able address the unique treatment-related responses which occur under immunotherapy. Patients tolerating immunotherapy with asymptomatic disease progression and/or mixed response should typically be treated based on irRC with continued treatment until progression is confirmed with a repeat scan. If progression is not confirmed then patient should continue on therapy.

As to which endpoint is believed to be the most important in evaluating an IO treatment for patients with aRCC, the subcommittee ranked the given endpoints in order from most to least importance: landmark OS, CR rate, median PFS, treatment free survival (TFS), OR rate, disease control rate (DCR), quality of life and cost effectiveness. Furthermore, when comparing VEGFR TKI/IO to IO/IO based combination therapies, the subcommittee agreed (74%) that 3-year landmark OS was the most relevant endpoint.

Regarding routine monitoring of patients, the majority of the subcommittee recommended standardized testing of LFTs (100%), TFTs (T4/TSH; 100%), CBC (94%) and LBC-glucose (83%). Other items recommended for routine monitoring included CPK/Troponin (33%), urinalysis (28%) and serum cortisol (22%). CPK/troponin testing is due to low risk, but serious consequences of myocarditis and myositis and the cortisol testing was recommended due to the potential impact of delayed detection of adrenalitis/ hypophysitis.

A subgroup analysis of a randomized phase 2 trial (NCT01354431) in patients with mRCC investigated the safety and efficacy of treatment with nivolumab beyond investigator-assessed first progression. Of 168 patients randomized to nivolumab, 154 experienced progression. Of those who progressed, 36 were treated beyond first progression, 26 were treated beyond first progression for ≤6 weeks, and 92 were not treated beyond first progression. Following initial progression, 69% of patients treated beyond progression experienced subsequent tumor reduction or stabilization in target lesion size with a low incidence of TRAEs. Results of this analysis demonstrated that a proportion of patients who continued treatment beyond RECIST-defined first progression experienced sustained reductions in tumor burden or stable disease, with an acceptable safety profile, noting this is a small and highly-selected subgroup of patients [44].

In CheckMate 025, 78% of patients treated with nivolumab progressed after initial treatment and 48% of these patients continued to be treated for ≥4 weeks after first progression. Nivolumab therapy was permitted after RECIST v1.1 defined progression if clinical benefit was observed [45]. 13% of patients who continued nivolumab treatment post-progression experienced ≥30% tumor burden reduction from the baseline assessment of first progression [45].

For an aRCC patient on anti-PD-1 monotherapy (e.g. nivolumab) who experiences RECIST-defined PD (e.g. in maintenance phase of ipilimumab/nivolumab or on nivolumab monotherapy) the majority of the subcommittee (75%) recommend to repeat scans in 4–12 weeks and to continue nivolumab if the patient is clinically well, until additional progression is documented.

Regarding how long to continue therapy in a patient with a CR or near CR after ipilimumab plus nivolumab induction and 6–9 months of maintenance nivolumab therapy, the subcommittee was split between recommending to stop at this point and monitor the patient versus treating the patient for a given number of cycles after best response before stopping. No members supported the notion of continuing therapy indefinitely. *Note: in Keynote-426, pembrolizumab was administered for a maximum of 35 cycles (2 years) [11].

Patient receives axitinib/IO combination therapy. At month 9 they have a CR/near CR/ over 80% response. In the absence of limiting toxicity, 94% of the subcommittee would be comfortable with stopping the IO component at 35 doses (2 year, however the subcommittee was split regarding whether they would be comfortable with stopping axitinib at any time: 56% would NOT recommend to stop axitinib maintenance therapy while 44% of the subcommittee would recommend stopping axitinib at some point.

In the absence of toxicity, the subcommittee recommended stopping IO therapy when patients demonstrate complete response (94%), confirmed or symptomatic progression (69%), and have received two years of therapy without PD (56%).

6. ***What is the role of biomarker testing in patients with aRCC?***

The majority of patients with aRCC will have disease progression on novel regimens, highlighting the importance of developing predictive biomarkers to better determine who will benefit from treatment with checkpoint blockade and/or an anti-PD1 in combination with VEGF inhibition and who might need an additional treatment approach.

### Literature review and consensus recommendations

#### PD-L1

Tumor expression of PD-L1 is utilized clinically as a biomarker of predicted response to ICIs in several solid tumors; however, the complexity of patient selection using PD-L1 IHC limits utility, and improved biomarkers and approaches are needed . Not only are there various assays and antibodies currently in use for measurement of PD-L1 expression, but there are also discrepancies as to how to define PD-L1 positivity ranging from positive PD-L1 expression of 1 to 50%. For some agents, the benefit appears to be enriched in PD-L1+ patients; however, because only 20–30% of RCC tumors express PD-L1 and tumor responses can be seen in patients with PD-L1- tumors, the number of responders with PD-L1- tumors can exceed those with PD-L1+ tumors. Therefore PD-L1 expression may be useful for patient stratification on clinical trials, but is not currently useful for treatment decisions and should not be routinely tested for. Biomarker data for CheckMate 025, CheckMate 214, Javelin Renal 101, and IMmotion150–151 are detailed in Table 4.

In CheckMate 025, tumor PD-L1 expression was analyzed (28–8 Dako assay) as either ≥1% or ≥ 5% of tumor cells. PD-L1 expression in this setting with nivolumab monotherapy was prognostic of poor outcome but not predictive of an overall survival effect, meaning nivolumab benefit was identified irrespective of PD-L1 expression [16, 47, 48].

Checkmate 214 analyzed the entire population as well as patients stratified by tumor PD-L1 expression. Longer PFS with the combination therapy relative to sunitinib was observed among patients whose tumors displayed ≥1% PD-L1 expression but not among those with < 1% PD-L1 expression. Longer OS and a greater ORR, on the other hand, were observed with nivolumab plus ipilimumab across all tumor PD-L1 expression levels, although the benefit was enhanced in the population with ≥1% PD-L1 expression (Table 4). Furthermore, CR rate was 16 and 7% in patients with > 1% PD-L1 and < 1% PD-L1 expression, respectively [9]. Similar to results of CheckMate 025, these results suggest that factors other than PD-L1 expression may be contributing to response and OS benefit from the combination therapy [16, 49]. Conversely, results of Keynote-426 demonstrated OS, PFS, and ORR benefits with the combination across all risk groups and regardless of tumor-based PD-L1 expression level (Table 4) [11].

In IMmotion150, patients were initially stratified by PD-L1 status, positivity being PD-L1 expression ≥1% (Ventana SP142 IHC assay) on tumor infiltrating immune cells. In patients with PD-L1+ tumors, PFS hazard ratios were 0.64 (95% CI, 0.38–1.08) and 1.03 (95% CI, 0.63–1.67), respectively [46, 50].

Subsequently, IMmotion151 met its primary endpoint of improved PFS in PD-L1-positive patients (≥ 1% tumor-infiltrating immune cells [IC]) treated with atezolizumab plus bevacizumab across all MSKCC risk groups compared to sunitinib [51]. For patients with PDL1+ tumors, PFS benefit was demonstrated in the atezolizumab-bevacizumab combination arm compared to sunitinib (mPFS: 11.2 vs. 7.7 mo; HR, 0.74; 95% CI, 0.57–0.96). In the same group, ORR was 43% and DOR was not reached for the combination arm vs 35% and 12.9 months for sunitinib-treated patients, respectively (Table 4).

In KEYNOTE-427, which examined pembrolizumab monotherapy in patients with accRCC, the response rate was higher in those with tumor-based expression of PD-L1 of ≥1% versus those with PD-L1 expression < 1% [24]. PD-L1 status was assessed using a combined positive score (CPS) method in which the number of PD-L1 staining cells of all types was divided by the total viable tumor cells and multiplied by 100 [52]. Specifically, in 46 patients with a CPS of at ≥1, confirmed ORR was 50.0%, and in 53 patients with a CPS < 1, it was 26% [24].

Eighty-nine percent of the subcommittee does not order any biomarker testing prior to treatment of patients with newly diagnosed ccRCC with immunotherapy. Two subcommittee members (11%) reported that they typically order tumor PD-L1 expression testing.

#### Gene expression signatures

In addition to analysis by PD-L1 tumor expression (Table 4), IMmotion150, IMmotion151 and JAVELIN Renal 101 trials conducted exploratory biomarker analyses to investigate the role of angiogenesis and T-effector gene expression signatures (GEs) in therapeutic outcomes. While the analysis from IMmotion150 suggested that tumor mutation and neoantigen burden were not associated with PFS, angiogenesis, T-effector/IFN-γ response, and myeloid inflammatory gene expression signatures were strongly associated with PFS within and across treatment groups, with a demonstrated improvement in PFS in T-effector high/Myeloid high tumors in the combination arm compared to atezolizumab monotherapy but not in the T-effect high Myeloid low arm. On the other hand sunitinib performed better in the angiogenic high than in the angiogenesis low population [46, 51, 53]. Such results are hypothesis-generating, although not yet impacting clinical practice.

#### Sarcomatoid histology

In CheckMate 025, many patients with poor risk features and/or sarcomatoid components demonstrated the greatest benefit with nivolumab [16, 47, 48].

An exploratory analysis of CheckMate 214 retrospectively evaluated the efficacy and safety of nivolumab plus ipilimumab vs sunitinib in patients with treatment-naive, advanced or metastatic clear cell RCC, with sarcomatoid features. Among patients with available tissue, tumor PD-L1 expression of at least 1% was observed in 50% of those with sarcomatoid RCC vs 27.5% of those without sarcomatoid features. Among patients with sarcomatoid RCC, ORR was 56.7% (95% CI, 43.2–69.4%) with nivolumab plus ipilimumab vs 19.2% (95% CI, 9.6–32.5%) with sunitinib (*P* < .0001). Significantly, the rate of CR was 18.3% with nivolumab plus ipilimumab vs 0% with sunitinib [54].

Patients with sarcomatoid histology with a good performance status were also included in the IMmotion151 study (Table 4). Interestingly, PD-L1 prevalence was higher in sarcomatoid tumors, compared to non-sarcomatoid tumors and angiogenesis gene expression was lower in sarcomatoid compared to non-sarcomatoid tumors (*p* = 4.73e-16) [51]. Particular benefit was observed in patients whose tumors demonstrated a sarcomatoid histology component.

As for first-line treatment for patients with sarcomatoid RCC irrespective of IMDC risk factors, 83% of the subcommittee recommend nivolumab plus ipilimumab combination immunotherapy while 11% recommend treatment with axitinib/pembrolizumab and 6% would recommend axitinib/avelumab.

7. ***What is the role of immunotherapy in non-clear cell pathology?***

RCC histologies other than clear cell, collectively known as non-clear cell renal cell carcinomas (nccRCC), account for 15–25% of primary kidney malignancies [55]. nccRCC comprises a diverse group of tumors including papillary, chromophobe, collecting duct, translocation, medullary and unclassified subtypes with pathologic and molecular features as well as clinical phenotypes distinct from ccRCC [56, 57]. Very few studies have sought to investigate whether immunotherapy is safe and effective in treating patients with advanced non-clear cell renal cell carcinoma (anccRCC).

### Literature review and consensus recommendations

While category 1 evidence does not exist regarding immunotherapy for patients with anccRCC, checkpoint blockade has demonstrated encouraging anti-tumor activity in this population, suggesting these patients should not be excluded from clinical trials or consideration for treatment with immunotherapy agents.

First-line pembrolizumab monotherapy was evaluated in a cohort of patients with anccRCC from KEYNOTE-427 (Cohort B). 165 treatment naïve patients with nccRCC, received pembrolizumab at 200 mg IV Q3W for 35 cycles, lasting about two years or until PD, unacceptable toxicity, or withdrawal. Confirmed histologies included: papillary 72% (*n* = 118), chromophobe 13% (*n* = 21), unclassified 16% (*n* = 26). 68% of patients were determined to be intermediate/poor IMDC risk, and 62% were PD-L1+ (combined positive score [CPS] ≥1 for PD-L1+). At a median follow-up of 11.1 months, 56% of patients discontinued anti-PD-1 therapy due to PD or clinical progression. ORR was 24.8% (95% CI, 18.5–32.2), with 8 [4.8%] CRs and 33 [20%] PRs. ORR (95% CI) was 25.4% (17.9–34.3) in patients with papillary histology tumors, 9.5% (1.2–30.4) in those with chromophobe tumors, and 34.6% (17.2–55.7) in those with unclassified nccRCC. ORR (95% CI) was 28.3% (16.8–42.3) for patients with favorable and 23.2% (15.8–32.1) with intermediate/poor IMDC risk and 33.3% (24.3–43.4) and 10.3% (3.9–21.2) for patients with tumor CPS ≥ 1 and CPS < 1 expression, respectively. Grade 3–5 TRAEs occurred in 11% of patients, while 6% discontinued due to TRAEs. Two patients died from TRAEs including pneumonia and cardiac arrest. Overall, pembrolizumab monotherapy in patients with anccRCC demonstrated promising antitumor activity, particularly in those patients with papillary or unclassified histology [58].

In a retrospective study, patients from six centers in the US who received at least one dose of nivolumab for non-clear cell mRCC (nccmRCC) were analyzed by patient characteristics and ORR according to RECIST v1.1 and TRAEs [59]. Of the 41 patients identified, tumor histologies included 16 papillary, 14 unclassified, 5 chromophobe, 4 collecting duct, 1 Xp11 translocation and 1 MTSCC (mucinous tubular and spindle cell carcinoma). Of the 35 patients evaluable for best response, 7 (20%) had PR and 10 (29%) had SD. The remaining 18 patients (51%) had PD (14 patients with radiographic PD and 4 patients with clinical PD) as best response. Observed PRs were in unclassified, papillary and collecting duct subtypes and 3 of the 4 patients with chromophobe histology had SD without observed response. Among patients who experienced an objective response to treatment, the tumor decreased in size by a mean percentage of 38%. Over the entire cohort, median follow-up was 8.5 months and median treatment duration was 3.0 months. Median PFS was 3.5 months and median OS was not reached., Median time to best response was 5.1 months, and median DOR was not reached (2/7 responders had PD during follow-up). TRAEs of any grade were noted in 37% of patients, with fatigue (12%), fever (10%) and rash (10%) being the most common. ICI treatment was suspended in 34% and discontinued in 15% of patients due to intolerance [59].

The subcommittee recommend IO-based therapy for first-line treatment of patients with papillary and unclassified RCC, specifically single-agent anti-PD-1 for either subtype with the additional treatment possibilities of ipilimumab/nivolumab combination therapy for the latter. The subcommittee was undecided between treatments with an IO-based monotherapy versus a TKI for first-line treatment of patients with chromophobe RCC. For patients with nccRCC whose disease has progressed on frontline VEGFR TKI, the subcommittee recommended anti-PD-1 monotherapy (nivolumab; 56%), or treatment with a TKI, specifically cabozantinib (22%).

8. ***Are there populations of patients with accRCC who should not receive immunotherapy (populations to consider/exclude from treatment)?***

Limited data exist on the safety and efficacy of checkpoint inhibitors in patients reliant on steroids or with underlying immune dysfunction. Current FDA approvals for combination therapy nivolumab plus ipilimumab and nivolumab monotherapy for patients with aRCC do not specify any eligibility restrictions such as underlying autoimmunity or other contraindications. Since both CTLA-4 and PD-1/PD-L1 pathways play vital roles in the systemic balance of the immune system, concerns arise in considering the possible toxicities linked with blocking associated signals and releasing the immune system in a patient whose immune system is already reacting to autologous organs/tissues. Additionally, concerns remain as to whether immunosuppressive therapies used to control a patient’s underlying symptoms would hinder any therapeutic benefit of checkpoint inhibition. The subcommittee discussed whether specific groups of aRCC patients would not be good candidates for IO treatment.

### Literature review and consensus recommendations

General patient inclusion and exclusion criteria for clinical trials investigating checkpoint blockade in aRCC were similar in first- and second-line immunotherapy-based clinical trials in other solid tumor settings. Relevant trial exclusion criteria included history of autoimmune disease (except controlled and treated hypothyroidism or type I diabetes mellitus), history of idiopathic pulmonary fibrosis, or pneumonitis, positive human immunodeficiency virus (HIV) test, active or chronic hepatitis B or C, prior allogeneic stem cell or solid organ transplantation, or current therapy with systemic corticosteroids (> 10 mg daily prednisone equivalent) or other immunosuppressive medications.

Of the general factors to consider when determining NOT to give nivolumab/ipilimumab combination therapy in patients with aRCC, the subcommittee felt that history of potentially life threatening AI condition and/or need for immunosuppressive therapy (94%), poor performance status (50%), and advanced patient age and IMDC risk stratification (39%) were the most influential.

Of the general factors to consider when determining NOT to give IO/TKI combination therapy in patients with aRCC, the subcommittee agreed that history of potentially life threatening AI condition and/or need for immunosuppressive therapy (72%) and recent history of cardiovascular co-morbidities (39%) were the most influential. Other factors felt to be important were advanced patient age (33%) and poor performance status (33%).

While very little data exists investigating the use of immune checkpoint blockade in patients with aRCC with preexisting autoimmune disorders, there have been some studies done in patients with melanoma examining treatment with ipilimumab or anti-PD-1 in this patient population. A retrospective review analyzed 30 patients with advanced melanoma and preexisting autoimmune disorders who received ipilimumab (mostly low-dose prednisone or hydroxychloroquine). In this study, the objective response rate was still 20%, including 1 CR - consistent with response rates reported in other populations. Additionally, 50% of patients experienced neither a flare of their autoimmune disease or grade 3 or higher irAE (27 and 33% of patients experienced each, respectively) [60]. Other studies reporting the use of ipilimumab therapy in patients with rheumatoid arthritis and multiple sclerosis (MS) demonstrated clinical activity with either no or only a mild increase in arthritic symptoms [61–63].

Retrospective reviews were also conducted in studies examining anti-PD-1 therapy in patients with advanced melanoma and either preexisting autoimmune disease and/or a history of irAEs during prior treatment with ipilimumab were treated with anti–PD-1 therapy. ORR was 33%, mirroring response rates seen in other populations. Although 30% of patients developed additional irAEs, the majority were easily managed [64].

94% of the subcommittee agreed that currently active autoimmune disease requiring medication would be considered a reason not to provide combination immunotherapy to an intermediate/poor risk patient with mRCC and 75% of the subcommittee recommend against treating patients receiving steroid dosing (for any reason) > 10 mg per day prednisone or equivalent. Fifty-six percent of the subcommittee, however, do not recommend excluding patients from treatment due to significant burden/pace of disease requiring rapid tumor burden reduction.

While IO therapies have become SOC for multiple malignancies patients such as those who present with a poor ECOG performance status or chronic viral infections [human immunodeficiency virus (HIV), hepatitis B (HBV) and hepatitis C (HCV)] were underrepresented in early clinical trials. A retrospective analysis investigated underrepresented patients treated with ICI-based monotherapy and combination therapies from January 2011 to April 2018, including patients with HIV, HBV/HCV, or a pre-treatment ECOG PS ≥2. Among patients with HIV, any grade and grade ≥ 3 irAEs were 24 and 10% with an ORR of 29%. In the HBV/HCV cohort, grade and grade ≥ 3 irAEs were 50 and 26% with an ORR of 21%. No viral reactivation was noted during ICI treatment. For patients with ECOG PS ≥2, the ORR was 14%. Any grade and grade ≥ 3 irAEs in this cohort were 20 and 4%. This data suggests that ICI therapy was not associated with significant safety concerns or lack of efficacy in the discussed populations [cite: ASCO abstract #2587, Neil J. Shah].

Specific to checkpoint inhibitor monotherapy, the subcommittee recommends NOT treating patients with aRCC who currently have active autoimmune disease requiring immunosuppressive medication (93%), or who require corticosteroid use > 10 mg/d prednisone equivalent (67%).

In regards to patients with advanced RCC who currently have controlled HIV and/or a history of hepatitis C or B infection, 89% of the subcommittee would NOT recommend AGAINST using checkpoint inhibitor-based therapy.

Specific to VEGFR TKI/checkpoint inhibitor combination therapy, the subcommittee recommends NOT treating patients with aRCC who currently have active autoimmune disease requiring immunosuppressive medication (87%), require corticosteroid use > 10 mg/d prednisone equivalent (53%), or who have poor performance status (20%).

While the use of corticosteroids for treatment of immune-related adverse events do not seem to affect therapeutic efficacy, the potential impact of baseline use of corticosteroids at the time of treatment initiation has had only limited study. In one study involving IO-naïve patients with NSCLC treated in two independent cohorts, ninety (14%) of 640 patients treated with anti-PD-(L)1 monotherapy were receiving the equivalent of ≥10 mg of prednisone daily at the start of checkpoint blockade. In both independent cohorts, baseline corticosteroids were associated with decreased ORR, PFS, and OS with PD-(L)1 blockade. Moreover, in a multivariable analysis of both cohorts, baseline corticosteroid use remained significantly associated with decreased PFS (hazard ratio, 1.3; *P* = .03), and OS (hazard ratio, 1.7; *P* < .001) [65].

9. ***Quality of life***

Many studies indicate significant quality of life improvements in cancer patients being treated with immunotherapies compared to TKIs. Quality of life issues include diarrhea, nausea, anxiety, and functionality to take part in a normal, everyday life. As patients with aRCC encounter many of these issues, the subcommittee discussed potential quality of life concerns pertaining to treatment with immunotherapies.

### Literature review and consensus recommendations

Category 1 evidence from aRCC trials demonstrated that while patients in the standard-therapy group reported a clinically meaningful deterioration from baseline and bothersome symptoms, more patients treated with nivolumab, nivolumab plus ipilimumab or atezolizumab plus bevacizumab reported more symptom stability or an improvement in health related quality of life.

In a secondary analysis of CheckMate 025 which compared health-related quality of life (HRQoL) between treatment arms and in relation to OS, 706 patients reported that treatment with nivolumab was associated with improvement in QoL, whereas those patients treated with everolimus experienced a deterioration in QoL. Assessments were made before any clinical activities and at the first two follow-up visits [66]. As assessed by the Functional Assessment of Cancer Therapy–Kidney Symptom Index–Disease Related Symptoms (FKSI-DRS; a disease-specific questionnaire), 55% of patients treated with nivolumab experienced clinically meaningful HRQoL improvement versus 37% in the everolimus arm (*p* < 0·001). Likewise, patients treated with nivolumab experienced an improvement from baseline in HRQoL beginning at week 20 (mean [SD], 0.6 [3.8], *p* = 0·031) through week 104 (3.5 [4.1], *p* = 0.001). Those treated with everolimus experienced a meaningful deterioration (*p* < 0.04) from baseline in HRQoL, starting at week 4 (− 1.5 [4.5], *p* < 0.001) through week 32 (− 1.1 [4.7], *p* = 0.019) and again from week 60 (− 1.6 [4.4], *p* = 0.016) through week 64 (− 1.5 [4.8], *p* = 0.040).

In CheckMate 214, statistically significant differences in the mean change from baseline were observed using FKSI-19, the revised FKSI questionnaire, total scores favoring nivolumab plus ipilimumab at all but two post-baseline time points through two years of follow-up (*P* < 0.05). Despite the prevalence of side effects and greater percentage of patients stopping treatment in CheckMate 214, patients who received the immunotherapy combination reported higher quality of life throughout the study. Specifically, despite the need for 29% of patients on the combination immunotherapy arm to receive immunomodulatory agents (high-dose glucocorticoids [≥40 mg of prednisone per day or equivalent]) to manage select treatment-related adverse events, the quality of life for patients on this treatment was superior to that for patients receiving sunitinib [9, 10].

In IMmotion151, PROs evaluated as exploratory endpoints found that patients on atezolizumab and/or atezolizumab plus bevacizumab maintained daily function with minimal symptom interference versus sunitinib, indicating improved quality of life on with ICI therapy versus TKIs [67]. Specifically, patients completed the MD Anderson Symptom Inventory (MDASI) and FKSI-19 questionnaires on days 1 and 22 of each 6 week treatment cycle, at the end of treatment, and during survival follow-up. Clinical survey topics included symptom burden (MDASI symptom severity and symptom interference with daily living) and bother from treatment side effects (FKSI-19 GP5 item). Patients receiving the combination therapy reported milder and more stable symptom severity, less interference, and better HRQoL compared to patients receiving sunitinib, who reported worsened interference on a more frequent basis. TTD in interference was also delayed in the combination arm versus sunitinib (median for atezolizumab plus bevacizumab was 11.3 months vs 4.3 months for sunitinib [HR 0.56; 95% CI 0.46, 0.68]). Finally, a greater proportion of atezolizumab plus bevacizumab-treated patients reported none or little bother due to treatment side effects vs sunitinib-treated patients [68].

## Conclusions

Immunotherapy has emerged as a new pillar of cancer treatment for patients with aRCC. With FDA approved immunotherapies for aRCC now in the frontline, the field is currently focused on which treatments to offer to which patients. Trials comparing front line options head to head linked to predictive biomarkers and using IO endpoints such as 3 year landmark OS and treatment free survival are needed to help rationally select between existing options for specific patient populations. Further, information is lacking on how best to manage patients on TKI/IO combination regimens and how to treat such patients if and when they exhibit disease progression. Similarly, advancement of other immunotherapies and strategies will be vital for continued progress in treating patients with this disease, as will overcoming challenges such as tumor immune resistance, immune escape and immune-related adverse events [69].

As a new standard in the field, every patient should receive an anti-PD-1-based therapy as initial treatment unless there is a specific contraindication to this approach. This is particularly true for patients with sarcomatoid histology, where the benefit of immunotherapy relative to VEGF TKI appears to be particularly strong. Recent data also supports treating patients with papillary and unclassified RCC with IO-based therapy in the first line setting. However, there remains a need for biomarkers to better predict patient response and to help decide the best treatment approach for each patient. Additionally, it remains to be determined whether new IO combinations including VEGFR TKIs will elicit properties of IO therapy, enabling the patient the ability to stop treatment with persistent benefit. Further studies need to address the question of who should receive combinations of IO with VEGFR TKI relative to who should receive nivolumab/ipilimumab combination therapy, how to best manage toxicity, and not only when to stop treatment but also what is the appropriate management for patients who have stopped therapy.

Figure 1 Immunotherapy treatment algorithm for advanced RCC based on current FDA approvals for first-line therapy. All treatment options shown may be appropriate. The final selection of therapy should be individualized based on patient eligibility and therapy availability based on the treating physician’s discretion. The goal of these algorithms are to provide advice as the consensus recommendations of the Subcommittee. 1) Baseline imaging considerations: CNS imaging is recommended for all patients; bone imaging should be considered for symptomatic patients. 2) “Need for systemic therapy” is defined as: not having low volume, slow growing disease. 3) “Candidate for immunotherapy” is defined as: i. Patients without active autoimmune conditions requiring immunosuppressive therapy or a history of potential life threatening autoimmune conditions; and ii. Patients without the need for corticosteroids to treat other conditions (e.g. brain metastases or spinal cord, compression, lymphangitic spread of tumor). 4) Refractory is defined as: disease progression by RECIST and/or irRECIST or clinical disease progression

## Supplementary information

Additional file 1. Subcommittee participant list.

## Data Availability

Not applicable.

## Abbreviations

AJCC: American Joint Committee on Cancer
ATC: anaplastic thyroid carcinoma
BICR: Blinded Independent Central Radiology
CAR: chimeric antigen receptor
CPS: combined positive score
CR: complete response
CT: chemotherapy
ECOG: Eastern Cooperative Oncology Group
EORTC QLQ-C30: European Organization for Research and Treatment of Cancer Quality of Life Questionnaire–Core 30 module
EQ-5D-3 L: European Quality of Life–5 Dimensions
FDA: U.S. Food and Drug Administration
HRQoL: health-related quality of life
IC: Investigator’s choice
ICI: immune checkpoint inhibitor
IHC: immunohistochemistry
irAEs: immune-related adverse events
irRC: immune-related response criteria
ITT: intent-to-treat
mAB: monoclonal antibody
MSI: microsatellite instability
NCCN: National Comprehensive Cancer Network
ORR: overall response rate
OS: overall survival
PD: progressive disease
PD-1: programmed cell death 1
PD-L1: programmed cell death ligand 1
PFS: progression-free survival
PR: partial response
PS: performance status
RECIST1.1: Response Evaluation Criteria in Solid Tumors v1.1
RT: radiotherapy
SBRT: Stereotactic Body Radiation Therapy
SD: stable disease
SITC: Society for Immunotherapy of Cancer
SOC: Standard of Care
TMB: tumor mutational burden
TRAE: treatment-related adverse event
