# Supplementary material for: The society for immunotherapy of cancer consensus statement on immunotherapy for the treatment of advanced renal cell carcinoma (RCC)
Source: J Immunother Cancer. 2019 Dec 20;7:354. doi: 10.1186/s40425-019-0813-8 (PMC6924043; doi:10.1186/s40425-019-0813-8)
Supplement: Supplementary file 1 — Additional file 1. Subcommittee participant list. [file 40425_2019_813_MOESM1_ESM.docx]

| Appendix I: Participant List | |
| --- | --- |
| Committee Chair and Co-Chair: |  |
| Michael B. Atkins, MD | Georgetown, Lombardi Comprehensive Cancer Center, Washington, DC |
| Brian I. Rini, MD | Cleveland Clinic Taussig Cancer Center, Cleveland, OH |
| Subcommittee Participants: | |
| Dena Battle | KCCure, Leesburg, VA |
| Robert A. Figlin, MD, FACP | Cedars-Sinai Medical Center, Los Angeles, CA |
| Daniel J. George, MD | Duke University School of Medicine, Durham, NC |
| Hans Hammers, MD, PhD | UT Southwestern, Dallas, TX |
| Tom Hutson, DO, PharmD | Baylor University Medical Center, Dallas, TX |
| Eric Jonasch, MD | MD Anderson Cancer Center, Houston, TX |
| Richard W. Joseph, MD | Mayo Clinic, Jacksonville, FL |
| David F. McDermott, MD | Beth Israel Deaconess Medical Center, Boston, MA |
| Robert J. Motzer, MD | Memorial Sloan-Kettering Cancer Center, New York, NY |
| Sumanta K. Pal, MD | City of Hope, Duarte, CA |
| Allan J. Pantuck, MD | UCLA School of Medicine, Los Angeles, CA |
| David I. Quinn, MBBS, PhD, FRACP | Norris Comprehensive Cancer Center, Los Angeles, CA |
| Virginia Seery, MSN, RN, ANP-BC | Beth Israel Deaconess Medical Center, Boston, MA |
| Martin H. Voss, MD | Memorial Sloan-Kettering Cancer Center, New York, NY |
| Christopher G. Wood, MD, FACS | MD Anderson Cancer Center, Houston, TX |
| Laura S. Wood, RN, MSN, OCN | Cleveland Clinic Taussig Cancer Center, Cleveland, OH |
